# Long-Circulating Hyaluronan-Based Nanohydrogels as Carriers of Hydrophobic Drugs

**DOI:** 10.3390/pharmaceutics10040213

**Published:** 2018-11-03

**Authors:** Chiara Di Meo, Mayte Martínez-Martínez, Tommasina Coviello, Marival Bermejo, Virginia Merino, Isabel Gonzalez-Alvarez, Marta Gonzalez-Alvarez, Pietro Matricardi

**Affiliations:** 1 Department of Drug Chemistry and Technologies, Sapienza University of Rome, P.le Aldo Moro 5, 00185 Rome, Italy; chiara.dimeo@uniroma1.it (C.D.M.); tommasina.coviello@uniroma1.it (T.C.); pietro.matricardi@uniroma1.it (P.M.); 2 Department of Pharmacokinetics and Pharmaceutical Technology, Miguel Hernandez University, San Juan de Alicante, 03550 Alicante, Spain; maytemartinezmartinez@hotmail.com (M.M.-M.); mbermejo@goumh.umh.es (M.B.); isabel.gonzalez@goumh.umh.es (I.G.-A.); 3 Instituto Interuniversitario de Investigación de Reconocimiento Molecular y Desarrollo Tecnológico (IDM), Universitat Politècnica de València, 46100 Burjassot, Spain; virginia.merino@uv.es; 4 Departament de Farmàcia i Tecnologia Farmacèutica i Parasitologia, Universitat de València, 46100 Burjassot, Spain

**Keywords:** nanohydrogels, hyaluronan, riboflavin, hydrophobic drugs, piroxicam, biodistribution, pharmacokinetic

## Abstract

Nanohydrogels based on natural polymers, such as polysaccharides, are gaining interest as vehicles for therapeutic agents, as they can modify the pharmacokinetics and pharmacodynamics of the carried drugs. In this work, hyaluronan-riboflavin nanohydrogels were tested in vivo in healthy rats highlighting their lack of toxicity, even at high doses, and their different biodistribution with respect to that of native hyaluronan. They were also exploited as carriers of a hydrophobic model drug, the anti-inflammatory piroxicam, that was physically embedded within the nanohydrogels by an autoclave treatment. The nanoformulation was tested by intravenous administration showing an improvement of the pharmacokinetic parameters of the molecule. The obtained results indicate that hyaluronan-based self-assembled nanohydrogels are suitable systems for low-soluble drug administration, by increasing the dose as well as the circulation time of poorly available therapeutic agents.

## 1. Introduction

Hyaluronic acid (HA) is a ubiquitous polysaccharide in the human body, crucial for many biological functions. HA and its derivatives have been clinically used as medical products for about thirty years [1]. More recently, HA has been employed as a building block for the preparation of new biomaterials with applications in pharmacy, tissue engineering and regenerative medicine [2,3,4,5]. Among the biomedical applications, HA is exploited for the development of drug delivery systems, thanks to its physicochemical properties, biodegradability, biocompatibility, low toxicity and ease of chemical modification [6]. In addition, its ability to bind CD44 and RHAMM receptors enables active targeting to certain tumors such as prostate, stomach or colon carcinoma, thus delivering the drugs into the cells. Moreover, increased clinical CD44 expression has been positively correlated to metastasis. This property represents a very useful strategy for a cancer-targeted drug delivery [7,8] and for the administration of chemotherapeutic drugs that are not capable of penetrating the cell membrane by themselves (i.e., without an appropriate carrier) [9].

One of the main drawbacks in the use of HA is its rapid elimination from the systemic circulation due to its recognition by the hyaluronic receptors of the reticuloendothelial system organs, such as liver and spleen, and its subsequent degradation by hyaluronidases [10,11]. To reduce the uptake and degradation of HA and to extend its circulation time, the polymer can be chemically modified or crosslinked to form nanohydrogels (NHs). By means of a crosslinking process, the HA chains undergo an alteration of their pharmacokinetic properties, thus prolonging their circulation time. In this sense, some studies on HA-based nanocarriers, specifically developed for targeting to tumor tissues [12,13,14], are recently published. The in vivo behavior of the HA-based nanocarriers shows an increase of the circulation time of such nanoparticles. Moreover, it is shown that HA nanoparticles can efficiently accumulate within tumors for at least two days, while unmodified HA is rapidly excreted from the body after IV injection [15].

Among the HA-based NHs, a specific type is represented by the physically “self-assembled” nanohydrogels, based on hydrophobically-modified HA, in which cholesterol or cholanic acid is mostly used as lipophilic moieties [12,16,17]. These NHs can be formed in aqueous environments by interactions among the hydrophobic moieties [18], promoted by several processes, such as ultrasonication or nanoprecipitation [17]. Recently, a new method able to obtain simultaneously the formation and the sterilization of self-assembling NHs by means of a standard autoclave cycle has been developed [19]. These NHs can act as carriers of both hydrophilic and hydrophobic drugs [9,19], as well as of several therapeutic proteins without affecting their activity [17,18].

Recently, a new type of amphiphilic HA derivative for NHs formation has been developed, using a riboflavin tetra-ester as hydrophobic moiety [20]. The obtained hyaluronate-riboflavin derivative (HA-Rfv) NHs show an improved stability with respect to HA-cholesterol based nanocarriers, along with an enhanced drug loading capacity; in particular, an excellent behavior is shown in loading hydrophobic molecules, thus leading to their enhanced apparent water-solubility and allowing to obtain aqueous formulations of insoluble drugs [20,21]. For this purpose, piroxicam is selected as model hydrophobic drug. Piroxicam is a nonsteroidal anti-inflammatory drug; it is commonly used for the treatment of pain, tenderness, swelling, and stiffness caused by osteoarthritis and rheumatoid arthritis. When administered by oral route, piroxicam can cause stomach and intestinal bleeding and ulcers, weakness, dizziness and other manifestations. Intravenous route allows overcoming the side effects but its poor solubility limits its use. In this sense, new formulations are required for the optimization of its dosage regimen and for the improvement of efficacy.

In this study, the in vivo behavior of HA-Rfv NHs was investigated, in terms of toxicity and biodistribution performances after systemic administration to healthy rats. Moreover, a model hydrophobic drug, the anti-inflammatory piroxicam, was efficiently loaded within the NHs and IV administered by tail vein to check the improvement of the pharmacokinetic parameters of the nanoformulation compared to those of the free drug.

## 2. Materials and Methods

### 2.1. Materials

Hyaluronate tetrabutylammonium salt (HA-TBA, M_W_ = 220 × 10^3^) was a Contipro product (Contipro a.s., Dolní Dobrouč, Czech Republic); riboflavin tetrabutyrate (Rfv) was purchased from TCI Europe N.V., Zwijndrecht, Belgium; and N-methyl-pyrrolidone (NMP), 1,6-dibromohexane, piroxicam, glycerol, dextrose monohydrate and rhodamine B isothiocyanate (rho) were obtained from Sigma-Aldrich (Schnelldorf, Germany). Furthermore, diazepam (Valium, Roche, Barcelona, Spain), ketamine (Ketolar, Parke-Davis, Madrid, Spain), atropine (atropine sulfate, Braun, Jaén, Spain), heparin (Heparin Leo 5%, BYK ELMU, Madrid, Spain) and medical grade silicone tubes (Dow Corning Silastic, Fisher Scientific, Madrid, Spain); internal diameter 0.5 mm, outer diameter 0.94 mm) were used. Other chemicals were reagent grade and were used without further purification.

### 2.2. Synthesis of Hyaluronate-Riboflavin Derivative

The synthesis of HA-Rfv was carried out according to the procedure described by Di Meo et al. [20] 2’3’4’5’-tetrabutyryl-3-(6-bromohexyl) riboflavin (Rfv-Br) was first obtained from 2’3’4’5’-tetrabutyl-riboflavin and 1,6-dibromohexane in dry N,N-dimethylformamide; then, in a typical reaction, the derivatization of HA-TBA with Rfv-Br was achieved by dissolving 300 mg of the polymer in 30 mL of NMP, followed by the addition of 119 mg of Rfv-Br previously solubilized in 3 mL of NMP to obtain a theoretical derivatization degree of 30% mol_Rfv_/mol_HA_ (mol of Rfv per mol of HA repeating units). The reaction was left under vigorous magnetic stirring for 48 h at room temperature in the dark; the mixture was then diluted with distilled water, transferred into a dialysis tube (cut-off 12,000–14,000) and dialyzed until water conductibity was about 1.5 μS. The final solution was frozen in liquid nitrogen and freeze-dried (yield > 80%). The obtained product was analyzed by FT-IR ATR (PerkinElmer Spectrum 100 Optica FT-IR spectrometer, Waltham, MA, USA) using a spectral resolution of 4 cm^−1^ with 64 co-added scans over a spectral range of 4000–600 cm^−1^. The derivatization degree of hyaluronan was assessed by UV-Vis spectroscopy (PerkinElmer LAMBDA 25 UV/Vis system, Waltham, MA, USA) using a calibration curve of Rfv at λ = 445 nm in the concentration range of 3.125–50 μg/mL in DMSO.

#### Nanohydrogel Formation and Characterization

For the nanohydrogel (NHs) formation, a HA-Rfv dispersion (1.5 mg/mL) in distilled water was autoclaved at 121 °C and 1.10 bar for 20 min (Juno Liarre autoclave, 230 VAC, 50/60 Hz, 12 A, 2000 W, Casalfiumanese (BO), Italy), thus obtaining the NHs suspension [14]. The particle dimensions and PDI, along with NHs formulation stability at 4 °C, were evaluated by means of dynamic light scattering (DLS) using a Submicron ParticleSizer Autodilute Model 370 (Nicomp International Inc., Orlando, FL, USA).

The critical aggregation concentration (CAC) of NHs was determined by analyzing a series of HA-Rfv dispersions (from 0.01 to 2 mg/mL) as previously described (autoclave at 121 °C, 1.10 bar, 20 min). The samples were investigated by means of DLS measurements evaluating the NHs size and the scattering intensity values [13]. The CAC value was determined by the intersection point of the two linear trends by plotting intensity vs. concentration.

For the long-term storage experiments, NHs samples were freeze-dried: a cryoprotectant solution (dextrose solution 20% *w*/*v* in water) was added to the NHs suspension (final dextrose concentration 1% *w*/*v*) and left under slow magnetic stirring overnight at 4 °C. The suspension was then frozen in liquid nitrogen and freeze-dried. The product was then re-suspended in a suitable volume of water (1.5 mg/mL) and vortexed for few minutes. The suspensions were analyzed by DLS to check the dimensions and the PDI of the re-formed NHs.

### 2.3. Synthesis of Fluorescent Derivatives

For in vivo biodistribution tests, HA-Rfv NHs and native HA were both labeled with the fluorescent dye rhodamine B isothiocyanate (NHs-rho and HA-rho).

For HA-rho synthesis, 160 mg of HA-TBA was dissolved in 64 mL of DMSO and 1.2 mL of rho solution (9 mg/mL in DMSO) was added; the reaction was left under vigorous stirring for 5 h at room temperature in the dark. Then, the product was extensively dialyzed and freeze-dried. Labeled NHs were obtained by adding 1.3 mL of rho solution (9 mg/mL in DMSO) to 107 mL of NHs suspension (1.5 mg/mL). The reaction was left under vigorous magnetic stirring for 5 h at room temperature in the darkness. Then, the solution was dialyzed and freeze-dried after dextrose addition as cryoprotectant, following the previously described procedure.

Fluorescent NHs-rho were re-suspended in water (1.5 mg/mL) and observed by a confocal microscope with laser scanning (Inverted Microscope Eclipse Ti-E, Nikon Instruments, Amsterdam, the Netherlands) with an oil immersion 60× objective.

### 2.4. NHs Formulation for In Vivo Tests

To obtain NHs formulation suitable for intravenous administration, the osmolarity and pH of the NHs suspensions were adjusted to 290 ± 10 mOsm/L and to pH 7.40 ± 0.05 by adding glycerol at the final concentration of 2.28 *w*/*v* and the required volume of 0.1 M phosphate buffer to pH = 7.40. The osmolarity of the preparations was measured using a Knauer K7400 (Berlin, Germany) osmometer and the stability of NHs suspension at 4 °C in these conditions of osmolarity and pH was monitored by DLS over a week.

### 2.5. Toxicity Studies

#### 2.5.1. Cell Culture Conditions

Jurkat T lymphocytes cell line (kindly provided by the Department of Pharmacology of the University of Valencia) were used between passages 4 and 30. Cells were grown and maintained in 75-cm^2^ flasks in RPMI 1640 + GlutaMAX medium (GibcoBRL) supplemented with 10% fetal bovine serum, 1% nonessential amino acids, 20 mM 2-[4-(2-hydroxyethyl)-1-piperazine]ethanesulfonic acid, penicillin (10,000 units/mL), and streptomycin (10,000 μg/mL) (EuroClone) at 37 °C in a humidified atmosphere with 5% CO_2_. All reagents were purchased by Sigma –Aldrich (Merck, Schnelldorf, Germany)

#### 2.5.2. Cytotoxicity Studies

Cytotoxicity assays were carried out on Jurkat T lymphocytes cells in 96-well microtiter plates with flat-bottomed wells by MTT assay. In total, 25,000 cells resuspended in 200 µL of culture medium were seeded into each well. Plates were incubated for 24 h at 37 °C in a 5% carbon dioxide atmosphere and then the medium was replaced with a fresh medium containing NHs at the final concentration of 225 µg/mL or 112 µg/mL per well. After 24, 48, or 72 h, 20 µL of a 5 mg/mL of MTT solution were added to each well. After a new incubation period (3 h), the dark blue precipitate was isolated by centrifugation and re-dissolved in DMSO. Absorbance measurements were taken at 570 nm with a Labsystems Multiskan EX plate reader (ThermoFisher, Madrid, Spain) and the values were corrected with reference to the measurement at 630 nm. Solvent alone and untreated cells were included as negative controls. Assays were carried out in quadruplicate. Data were expressed as a percentage of the absorbance of the untreated control.

### 2.6. In Vivo Tests: Biodistribution and Toxicity Assay

In this assay, the biodistribution of HA and HA-Rfv NHs in rats was evaluated by determining the fluorescence emission of rhodamine in several vital organs following intravenous administration. Moreover, blood analysis and liver biopsy were performed to evaluate the potential toxicity.

A solution of HA-rho in water (3 mg/mL) and a suspension of NHs-rho in water (concentration equivalent to 4.5 mg/mL of NHs) were prepared. Concentrations were different for an optimized fluorescence visualization because each condition was independently evaluated. The osmolarity and pH of both formulations were adjusted to suitable values for intravenous administration (290 mOsm/L, pH 7.4) by the addition of glycerol and phosphate buffer as previously described.

Experiments were performed according to the protocol approved by the Ethics Committee (UMH-DI-MBS-02-15). Male Wistar rats weighing 280–300 g were anesthetized and were cannulated in the jugular vein 24 h before each experiment, using a previously described technique [22,23]. The animals were divided into 9 groups (*n* = 4): 4 groups received HA-rho, 4 groups received NHs-rho, and 1 group received saline serum as a control to study the baseline fluorescence of organs in absence of rhodamine. To facilitate the administration, rats were immobilized with the aid of a restrainer and 1.0 mL of the formulation tested was administered by intravenous injection into the tail vein; the rats were then returned to their cages with food and water ad libitum. Animals receiving the formulations were sacrificed at 5, 10, 24 and 48 h after administration, depending on the group.

At the predefined times, the animals were sacrificed by CO_2_ inhalation and immediately after the thoracic cavity and the upper abdominal cavity were opened to exsanguinate. First, a blood sample was taken from the heart. Then, a needle connected to a saline serum bag was inserted into the left ventricle and the right atrial appendage was cut, allowing drainage of all organs and exsanguination. At the end of this process, the following organs were excised: spleen, brain, heart, liver, lungs and kidneys. The blood was centrifuged at 8000 rpm for 10 min and the supernatant (plasma) was taken. The organs were weighed and homogenized. A sample of the hepatic tissue was collected before homogenization and was placed into formol 20% to preserve it. Hepatic biopsies of these tissues were carried out to by ACV LAB—Laboratorio de Analítica Clínica Veterinaria S.L. to analyze the potential changes due to NHs accumulation.

Standard blood test parameters and hepatic profile specific parameters (focusing on cholesterol triglyceride and hepatic enzymes levels) were determined at 24 h by ACV LAB—Laboratorio de Analítica Clínica Veterinaria S.L. Parameter values of treated and non-treated rats obtained with the different routinely laboratory techniques were compared using t-student to detect the existence of significant differences at the 0.05 probability level. The Levene’s statistic was calculated to test the homogeneity of variances and, depending on the result, post hoc tests were applied to determine statistical significant difference among groups. The statistical analysis was made using SPSS, Version 22 (licensed to University of Valencia).

For the biodistribution analyses, plasma and 0.5 g organ homogenates samples were placed in well plates and fluorescence images of rhodamine were taken by the InVivo FX Pro instrument (Bruker BioSpin, Ettlingen, Germany) (excitation wavelength of 530 nm, emission wavelength of 600 nm; software parameters: 40 s, 120 FOV, 5 mm focal plane). To remove the fluorescence baseline signal from the organs and plasma, control fluorescence signal (rats treated with saline serum) was also evaluated.

The intensity signals were analyzed using ImageJ**^®^** software (ImageJ2 version, free software, National Institute of Health, Bethesda, Maryland, USA). For each sample, the recorded fluorescence was corrected with the fluorescence baseline of each organ or plasma and multiplying by the weight of each organ or whole blood. The obtained results in liver and plasma were evaluated using the t-student statistical test for independent samples with a 95% confidence interval.

### 2.7. Model Drug Loading into NHs

The anti-inflammatory drug piroxicam was chosen as a model hydrophobic molecule. The loading process was carried out according to the previously reported procedure [19,20] that allows the simultaneous formation of NHs and their loading. Briefly, a HA-Rfv dispersion in distilled water was prepared (1.5 mg/mL, 30 mL) and left overnight stirring at room temperature. A stock solution of piroxicam in acetone (20 mg in 5 mL) was separately prepared and vacuum evaporated in a glass bottle leading the formation of a thin film of drug. The polymer dispersion was then added to the bottle containing the drug film and let it hydrating for 1 h, then the sample was autoclaved (121 °C, 1.10 bar, 20 min) to allow the formation of NHs and the loading of the hydrophobic drug. Once the samples were cooled down, they were centrifuged at 3000 rpm and 20 °C for 10 min (Ultracentrifuge Sorvall WX ULTRA 80 Thermo Fisher Scientific, Madrid, Spain) to precipitate the unloaded drug.

The supernatant, containing the drug-loaded NHs, was analyzed by DLS to determine the dimensions of the particles. The unloaded piroxicam, separated by centrifugation, was solubilized in ethanol and quantified by HPLC using a Varian System, model 210, equipped with a Varian ProStar 325 UV-Vis detector and a Knauer C18 column, 250 mm × 4.6 mm (Eurospher II, 100-5 C18). The mobile phase, a mixture of aqueous 0.1% *v*/*v* trifluoroacetic acid:acetonitrile 50:50 *v*/*v*, was used applying an isocratic flux of 1 mL/min; the injection volume was 20 μL and the detection was performed at the wavelength of 356 nm. The quantification of the unloaded drug was evaluated by means of a calibration curve obtained with piroxicam solutions with a range of concentrations from 15.63 μg/L to 0.25 mg/mL in ethanol (*r*^2^ = 0.9994).

The difference between the initial drug amount (*m_i_*) and the unloaded drug (*m_u_*) gives the encapsulation efficiency (*%EE*), according to Equation (1):

(1) $$\;\%EE=\;\frac{m_{i}-m_{u}}{m_{i}}\;\times \;100\;$$

whereas the drug loading percentage (*%DL*) of the formulation was calculated using Equation (2):

(2) $$\;\%DL=\;\frac{m_{l}}{p+m_{l}}\;\times \;100\;$$

where *m_l_* = *m_i_* − *m_u_* is the amount of loaded drug, and *p* is the amount of NHs used.

### 2.8. Piroxicam Pharmacokinetics

To determine piroxicam pharmacokinetic parameters, the drug was administered both in solution and encapsulated in NHs. Male Wistar rats 2–3 months old and 300 ± 10 g of weight were used and divided into two groups (*n* = 8) depending on the administered formulation: the first one received 1.25 mL of a piroxicam solution 0.29 mg/mL solubilized in PBS:propilenglycol (50:50) and the second group received 1.25 mL of an aqueous suspension of NHs loaded with piroxicam (equivalent to 0.29 mg/mL of piroxicam). The osmolarity and pH of the suspension were adjusted as described above (290 mOsm/L, pH 7.4).

To perform in vivo assays, 24 h before the administration, a silicone catheter was implanted in the jugular vein of the rats, following a previously validated technique [23]. Blood samples (0.2 mL) were withdrawn, at previously established sampling times, using heparinized syringes and the extracted volume was replaced with heparinized saline serum (1 IU/mL). Samples were immediately centrifuged (9600 g, 10 min) to separate plasma; proteins were precipitated adding cold methanol 2:1 and by centrifugation (9600 g, 15 min). Samples were analyzed by HPLC using an Alliance HPLC System (Waters, Barcelona, Spain). The pharmacokinetic analysis was carried out on mean experimental plasma concentration values versus time. The total area, AUC_0-∞_, was calculated by the trapezoidal rule for the data up to the last sampling time and adding the value of last experimental drug concentration divided by terminal mono-exponential rate constant (Cp/k). To calculate clearance and volume of distribution, two models of increasing complexity (one-compartment and two-compartment models) were fitted with experimental data. To select the model that better described obtained data, we evaluated R-squared value of the correlation between predicted versus observed values and sum of squared residuals (SSR). An improvement of the suitability of SSR value by a more complex model was statistically assessed with a Snedecor’s F test. The more complex model was accepted at a significance value *p* < 0.05. Mean values and standard deviation of the parameters calculated with best model in both groups (piroxicam in nanoparticles or as a free drug) were compared with two-tailed Student *t*-test. The statistical analyses were done with the statistical package SPSS (Version 22, IBM United States) licensed to Universidad Miguel Hernandez).

## 3. Results

### 3.1. Synthesis of the HA-Rfv Derivative and NHs Formation

**Figure 1:**
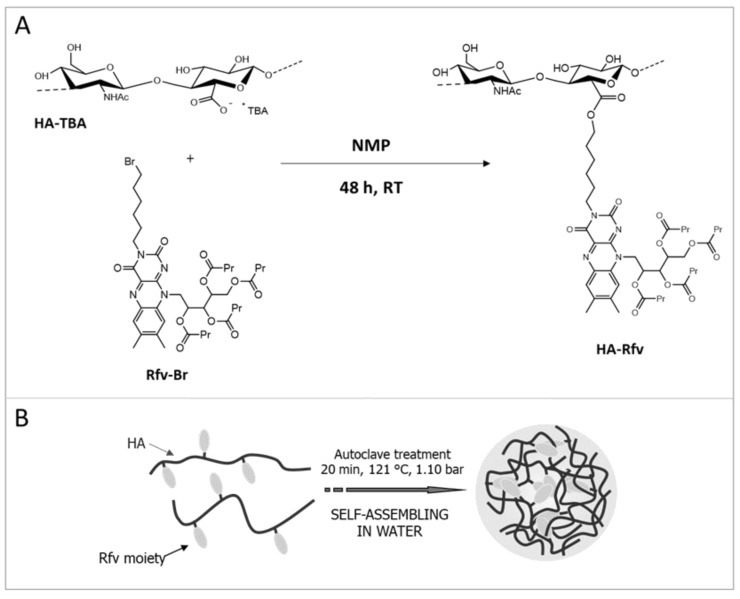
Scheme of HA-Rfv synthesis (**A**); and of NHs formation by autoclave treatment (**B**).

By the reaction of HA-TBA with Rfv-Br, an amphiphilic polymer was obtained [20], capable of self-assembling in aqueous environment under suitable conditions, as nanoprecipitation, ultrasound or autoclave treatments [17,18,19,20,21], thus forming NHs. For the synthesis, the use of the tetrabutiryl-Rfv reagent is due to the low solubility of not-esterified riboflavin in both aqueous and organic solvents; similarly, the TBA^+^ salt of HA was used to carry out the nucleophilic substitution in an aprotic polar solvent (DMSO). As shown in Figure 1A, Rfv-Br reacts with the carboxyl groups of HA-TBA leading to the formation of ester bonds. The obtained HA-Rfv polymer, recovered as a yellow freeze-dried product, is a partial ester of HA: FT-IR analysis carried out in ATR mode on the dried product, as reported in Figure S1, shows a notable stretching absorption at 1744 cm^−1^ due to the C=O stretching of the ester groups formed between HA and Rfv, and of those of tetrabutyl moieties on the Rfv molecules. The derivatization degree of the polymer was estimated to be 24% mol_Rfv_/mol_HA_ by means of UV-Vis spectrometry in DMSO (Figure S2).

HA-Rfv presents physicochemical characteristics very different from those of the native polymer. Water solubility is appreciably reduced by the hydrophobic Rfv moieties, thus the amphiphilic derivative, when autoclaved, is able to self-assemble in nanostructures [20]. The autoclave process allows the spontaneous formation of NHs by self-assembling of the HA-Rfv suspension due to the high pressure and temperature conditions that promote intra- and inter-molecular interactions among the hydrophobic groups of the HA-Rfv chains [19] (Figure 1B). FT-IR ATR analysis on dried NHs was repeated to verify the stability of the system and the preservation of the polymer structure after the autoclave treatment (Figure S1).

The NHs showed a mean diameter of 200 ± 30 nm with a low PDI (0.22 ± 0.04). For long-term storage of NHs, a freeze-drying process was developed and optimized [17]. Actually, the NHs aqueous suspensions underwent several degradation processes, such as aggregation and hydrolysis, and, when loaded, they can lose the entrapped drug. To obtain a suitable formulation, it should be kept in mind that the elimination of water by lyophilization induces a phase separation due to the formation of a frozen solution and a cryoconcentrated suspension where the NHs and the other compounds used in the formulation are present. This high concentration of NHs can induce aggregation and, in some cases, their irreversible collapse; in addition, the crystallization of water causes mechanical stresses that can modify the NHs structure. For these reasons, before the freeze-drying process, dextrose, as cryoprotectant, was added to the formulation (C_fin_ = 1% *w*/*v*); the dextrose molecules are able to form a compact matrix around NHs, thus preventing their aggregation and modification, induced by the mechanical stresses.

No significant changes in size or in PDI values after dextrose addition, and after freeze-drying and re-suspension in water were evidenced (Figure S3 in Supplementary Materials). Thus, it can be stated that lyophilization carried out in the presence of a cryoprotectant is a suitable method for NHs conservation. In addition, NHs lyophilized and re-suspended in water were stable for a week at 4 °C (Figure S4 in Supplementary Materials).

**Figure 2:**
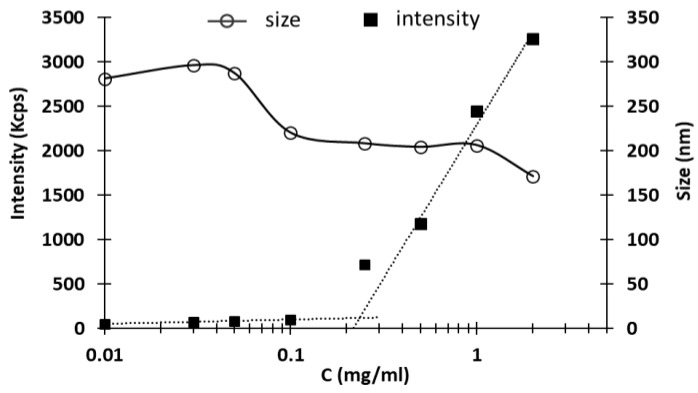
Intensity of light scattering and size of the NHs as a function of HA-Rfv concentration.

Another investigated parameter of NHs suspensions was the critical aggregation concentration (CAC). The CAC is defined as the minimum concentration of polymer at which NHs start forming by aggregation of the polymer chains. The DLS results, obtained with different samples, are reported in Figure 2: at low concentrations, few nanosized structures were detected, while the scattering intensity recorded at c = 0.25 mg/mL starts to increase exponentially due to the particulate dispersion, indicating the beginning of NHs formation [18]. The CAC value, expressed as the concentration of HA-Rfv evaluated at the intersection between the two linearized regions, is 234 μg/mL.

### 3.2. Synthesis of Fluorescent Derivatives

For the in vivo biodistribution tests, it was necessary to mark the NHs by means of a fluorescent molecule to quantify the product in the various organs and tissues. At the same time, HA was also labeled with the same dye for the in vivo comparison of the behavior of the two different materials, HA and HA-Rfv.

Rhodamine B isothiocyanate was chosen as fluorescent marker: the reaction between the isothiocyanate functional group of the dye and the primary hydroxyl group of the hyaluronate, both in the native polymer and in the NHs, leads to the formation of a thiocarbamate bond, as shown in the Figure S5A,B.

**Figure 3:**
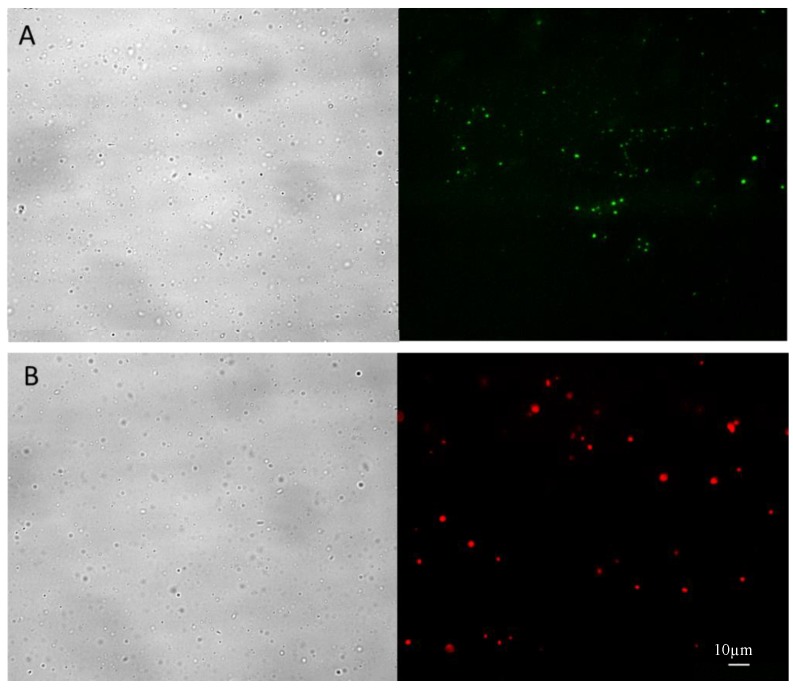
Optical (**left**) and fluorescence (**right**) microscopy images of: (**A**) unlabeled NHs; and (**B**) rhodamine-marked NHs (NHs-rho). Interval is 10µm

The purified NHs-rho were characterized after freeze-drying and re-suspension in water by optical and fluorescence microscopy. As shown in Figure 3A, the unlabeled NHs show the intrinsic fluorescence of the riboflavin molecule (green fluorescence), whereas the marked NHs show the typical intense red fluorescence of rhodamine (Figure 3B), without noise background due to fluorescent molecules not linked to the NHs.

### 3.3. Toxicity Studies

#### 3.3.1. Cell Toxicity Studies

NHs suspensions were examined for their potential cytotoxic activity. The effect of the formulation was tested on Jurkat T lymphocytes cells using the colorimetric MTT assay to determine cell viability after the cell exposition to various formulation concentrations (225 or 112 µg/mL), for 24, 48, 72 h. Table 1 shows the percentage of cell survival after formulation exposition, taking as control value the not exposed culture.

#### 3.3.2. In Vivo Assays

To assess the in vivo toxicity of the NHs, blood tests and hepatic biopsy were chosen as representative. After administration of the NHs or saline serum by tail vein, standard blood analyses and hepatic profile parameter values were determined and liver biopsies were performed to assess the effect of the NHs. Table 2 shows the parameter values obtained after administration of NHs or saline serum (control values of non-treated rats). Results show that there are no statistically significant differences (*p* < 0.05) in all compared parameters.

Because of the main accumulation of HA and NHs in the liver, data for hepatic profile were collected and the results are summarized in Table 3. After analyzing the hepatic profile parameters, the results determined that there are no statistically significant differences between control group animals and those exposed to NHs administration.

Results from the blood tests and the hepatic biopsies indicate that NHs did not induce toxicity or hepatotoxicity in the tested animals.

T-student was used to detect the existence of significant differences at the 0.05 probability level and there were no significant differences between hepatic profile values obtained in both groups (Table 3). The Levene’s statistic was calculated to test the homogeneity of variances and, according to these results, a parametric test was applied to determine statistical significant difference between groups in all parameters. The statistical analysis was made using SPSS, Version 22 (licensed to University of Valencia).

**Figure 4:**
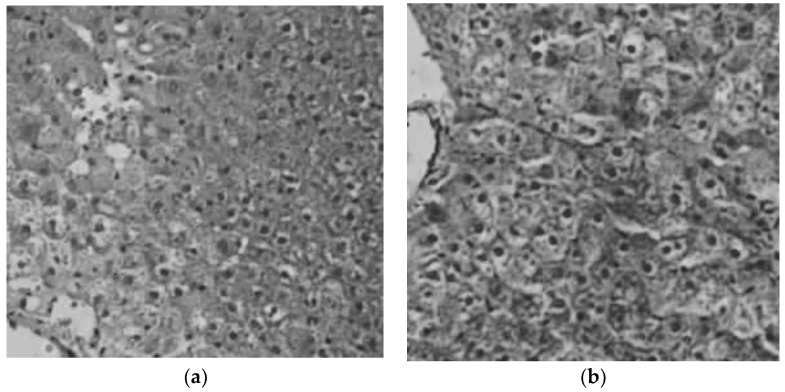
Hepatic biopsies images: (**a**) untreated animal from de control group; and (**b**) animal treated with NHs.

Figure 4 shows an example of an image taken from an animal of the control group and another image from the liver of a rat treated with the NHs. Hepatic biopsies indicate that, any accumulation of pigment in the hepatocytes, neither relevant inflammatory changes in the hepatic parenchyma nor space portal were determined in both groups, the group of animals that were administered saline serum and the group that was administered the formulation of NHs.

### 3.4. Biodistribution of HA and NHs

The percentage of accumulation of NHs and HA polymer in organs was evaluated by determining the fluorescence emitted by plasma and vital organs studied at different times after intravenous administration.

Obtained results indicate that the fluorescence signal is almost null (less than 2%) in lungs, spleen, heart and brain at any time, indicating that neither the polymer nor the NHs accumulate in these organs. As expected, the liver is the main organ of biodistribution and constitutes 98.2% and 85.5% of the fluorescence recorded at 48 h for HA and NHs, respectively (Figure S6 in Supplementary Materials).

Concerning kidney data, it is observed that NHs are eliminated by urinary excretion between 24–48 h after administration (5.7–9.1% of total fluorescence). However, the kidney fluorescence signal in rats receiving the labeled polymer is practically nil at all times.

Finally, it is interesting to note that the NHs have a longer circulating time in blood, since at 24 h the fluorescence signal is 41.3% and at 48 h it is 3.25%, in contrast to the HA polymer that recorded intensity values lower than 1% at both times.

**Figure 5:**
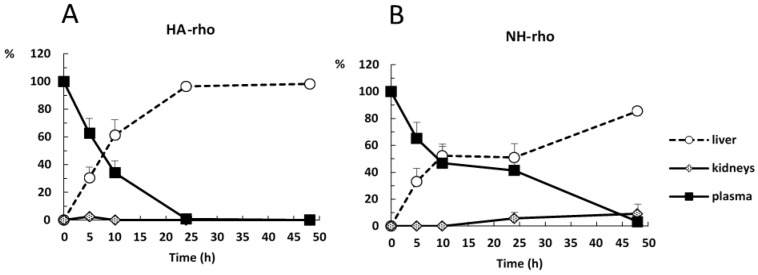
Evolution of relative accumulation in liver, plasma, and kidney of: (**A**) HA; and (**B**) NHs.

Figure 5 shows the evolution of the accumulation of the polymer and the NHs in plasma, liver and kidneys. At time zero, it is assumed that the administered formulation is entirely in plasma and as time progresses it is distributed mainly to liver.

**Figure 6:**
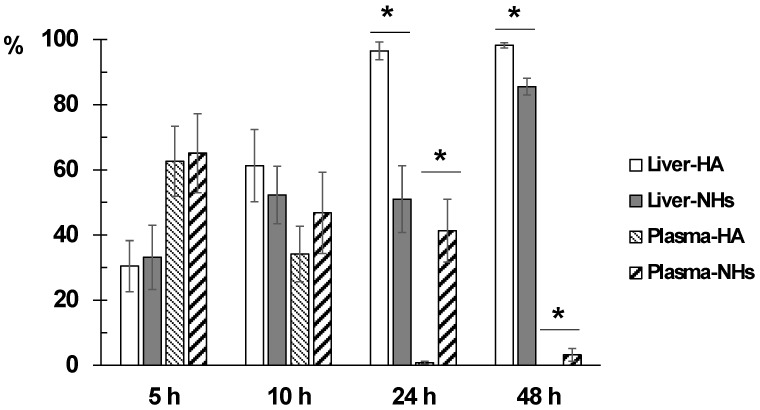
Comparison of the evolution of the percentage of accumulation of HA and NHs in liver and plasma. * indicates tatistically significant differences.

Figure 6 shows the evolution of disappearance in blood and the accumulation in liver of both formulations. The difference between the percentage of distribution of NHs and polymer in liver and plasma is statistically significant at 24 and 48 h after administration. The retention time of the NHs in plasma is significantly higher than that of HA.

The results are consistent with those previously published [15,24]. The reticuloendothelial system rapidly removes HA from the bloodstream, the polymer is extensively metabolized in hepatic endothelial cells and its metabolites are reused in the body. Kidney uptake is low, and the lower is the molecular weight of the polymer, the lower is the uptake in this organ [10]. HA-based NHs are accumulated mainly in the liver, while the accumulation in other organs of the reticuloendothelial system (kidneys and spleen) is variable [15,24]. Finally, in other organs, such as lungs, brain and heart, the biodistribution of NHs is minimal [24].

Based on the obtained data, it is concluded that the main advantage of the NHs formulation is that they have a significantly longer circulation time in blood compared to the starting polymer, since the percentage of polymer is less than 1% at 24 h compared to 41.3% of NHs at this time. This is an important goal to obtain stable plasma levels for longer time of low solubility drugs such as piroxicam.

### 3.5. Drug-Loaded NHs Formulation

Piroxicam, chosen as model hydrophobic drug, was loaded into NHs during the autoclaving process [19] by hydrating a preformed drug film and obtaining the formation and the loading of NHs in one single step. The polymer concentration, as well as the initial amount of drug, were previously optimized (data not shown) to obtain the best formulation in terms of NHs size and PDI, drug encapsulation efficiency (%EE) and loading percentage (%DL).

The best formulation was obtained by using a polymer concentration of 1.5 mg/mL with a feed of piroxicam in the ratio 1:2.25 with respect to polymer amount. In these conditions, NHs with suitable dimensions (215 ± 10 nm, PDI = 0.22 ± 0.02), with the highest %EE = 49 ± 5, %DL = 18 ± 1%, and Cpir = 0.32 ± 0.04 mg/mL, were obtained.

To obtain adequate osmolarity and pH values for the intravenous administration, a suitable amount of glycerol (2.28% *w*/*v* for osmolarity adjustment to 290 ± 10 mOsm/L) and of phosphate buffer solution (0.1 M, pH = 7.4, for adjustment to pH 7.40 ± 0.05, final concentration 0.01 M) were added to the formulation, after freeze-drying and re-suspension in water Table S1 (Supplementary Materials) shows how the size and PDI values of NHs were not influenced by the medium variation, and Figure S7 reports the stability of the final formulation over time.

### 3.6. Piroxicam Pharmacokinetics

**Figure 7:**
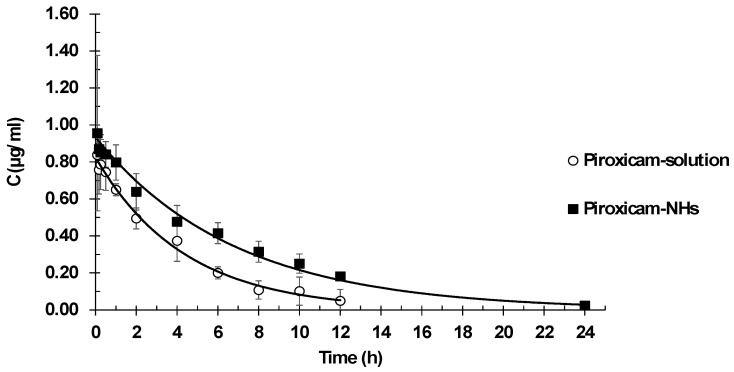
Plasma concentration piroxicam and piroxicam-NHs after IV administration.

Pharmacokinetic plasma profiles were determined after intravenous administration of a piroxicam solution or piroxicam encapsulated in NHs. Plasma drug concentration versus time are reported in Figure 7. Pharmacokinetic parameter values are listed in Table 4.

The best fit model was one-compartment model. It showed lower values of goodness of fits parameters in comparison with two-compartment model: minor SSR value (1.47 × 10^−3^ vs. 1.54 × 10^−3^) and less AIC value (−67.26 vs. −63.84). The one-compartment model fitted experimental data significantly better than the two-compartment model. The one-compartment produced lower SSR (1.47 × 10^−3^ vs. 1.54 × 10^−3^) and AIC (−67.26 vs. −63.84) values.

The results show the suitability of NHs as vehicles for intravenous administration of drugs, allowing modifications of the piroxicam pharmacokinetic behavior. The main difference in pharmacokinetic parameters between piroxicam in solution and piroxicam embedded within the NHs is clearance, which showed a 30% reduction. This reduction in clearance allows maintaining high concentrations of drug for a longer period, which implies a prolonged exposure to the drug. This is confirmed by the AUC values, as indicators of drug exposure, with piroxicam loaded NH presenting AUC values 43% higher than those of piroxicam in solution. Since clearance determines administration frequency, the detected lower clearance should eventually allow a reduction of the number of daily doses. On the other hand, piroxicam is a low solubility drug and, consequently, for its IV administration, a high concentration of propylene glycol as a cosolvent should be used. High levels of cosolvents are not always adequate in formulations for human use. In this sense, NHs provide a good option to overcome this inconvenience leading to an improved anti-inflammatory effect.

## 4. Conclusions

In this work the formation of NHs from an amphiphilic polymer obtained by hydrophobic modification of HA with a riboflavin derivative was studied. This polymer is able to self-assemble and spontaneously forms NHs when autoclaved in appropriate conditions. Furthermore, during the autoclave process, it is possible to obtain, at the same time, sterile and drug-loaded NHs. Piroxicam, chosen as model hydrophobic drug, was loaded in the NHs and the new system could act as drug carrier. NHs suspensions, both drug loaded and non-loaded, showed an appropriate size (about 220 nm) with a low PDI value (<0.25) and a noticeable stability. Moreover, it was shown that freeze-drying, after the addition of the selected cryoprotectant, is a suitable method for the long-term conservation of NHs without variation of their dimensions.

Among the most important results, it must be evidenced that NH-Rfvs are not toxic, both in vitro and in vivo; moreover, blood and hepatic analyses demonstrate their biocompatibility and tolerability. This system is an excellent option for the encapsulation of low solubility drugs which allows administering significantly higher doses of the drug with respect to the free drug. 

After 24 h from the intravenous administration, more than 40% of the NHs were still in the blood circulation, compared to less than 1% of free HA. Furthermore, NH-Rfvs allow modifying the pharmacokinetic parameters of the loaded active ingredient, leading to a 30% clearance reduction and a 43% AUC increase, compared to the drug solution administration. These innovative drug administration systems appear to be quite fundamental in situations where it is important to prolong circulation time in the body, as in the case of short biological half-life drugs.

## Figures and Tables

**Table 1:** Toxicity results expressed as percent of Jurkat T lymphocytes living cells.

	% Living Cells		
	24 h	48 h	72 h
Control without NHs	100	100	100
NHs 112 µg/mL	100.0 ± 5.2	97.2 ± 4.3	100 ± 6.3
NHs 225 µg/mL	99.3 ± 4.8	100 ± 2.4	99.3 ± 5.1

**Table 2:** Values of the standard blood test parameters.

	Control			NHs Administered		
	MEAN	SD	CV	MEAN	SD	CV
White blood cells (G/L)	2.31	0.58	25.22	3.30	0.46	13.81
Red blood cells (T/L)	8.58	0.52	6.09	7.04	1.12	15.88
Hemoglobin (g/dL)	15.23	0.70	4.61	13.00	1.44	11.09
Hematocrit (%)	41.87	0.57	1.36	37.93	1.11	2.92
Mean corpuscular volume (fL)	48.90	2.52	5.16	49.07	2.35	4.78
Mean corpuscular hemoglobin (pg)	17.73	0.32	1.81	18.60	1.31	7.05
Mean corpuscular hemoglobin conc (g/dL)	36.37	1.23	3.39	37.87	2.71	7.17
Red blood cells distribution width	19.27	1.01	5.25	15.97	2.14	13.38
Platelets (G/L)	513.00	141.67	27.62	396.33	68.53	17.29
Mean platelets volume (fl)	6.90	0.20	2.90	7.47	0.12	1.55
Plateletcrit (%)	0.34	0.11	33.78	0.24	0.05	18.53
Platelet aggregates	None	None	None	None	None	None

**Table 3:** Hepatic profile values obtained in both groups of rats in blood samples (*n* = 4).

	Control			NHs Administered		
	Value	SD	CV	Value	SD	CV
Biliars Acids	31.00	5.66	18.25	24.33	1.15	4.75
Urea	41.00	4.24	10.35	37.67	7.09	18.84
Total Cholesterol	108.50	34.65	31.93	108.00	14.11	13.06
Creatinine	0.50	0.01	2.83	0.50	0.03	6.39
Triglycerids	82.50	27.58	33.43	74.33	23.76	31.96
Total Proteins	6.65	0.07	1.06	6.33	0.23	3.65
Total Bilirubin	0.10	0.01	7.44	0.14	0.05	35.80
CPK	332.50	146.37	44.02	376.33	94.11	25.01
Phosphatase alcaline	153.50	7.78	5.07	122.00	20.88	17.12
GGT	<5	<5	<5	<5	<5	<5
GOT	100.00	5.66	5.66	106.00	11.14	10.51
GPT	32.50	4.95	15.23	33.67	3.06	9.07
LDH	462.00	124.45	26.94	435.67	98.65	22.64
Cholinesterase	0.24	0.00	0.00	0.28	0.04	14.61
Alpha fetoprotein	1.70	0.14	8.32	1.40	0.10	7.14
Albumin	2.77	0.15	5.37	2.18	0.12	5.65
Alpha1 globulin	1.90	0.10	5.21	1.74	0.09	5.35
Alpha2 globulin	0.61	0.01	2.32	0.89	0.11	12.73
Beta globulin	1.10	0.01	0.65	1.26	0.02	1.65
Gamma globulin	0.28	0.02	7.71	0.25	0.05	19.87
Albumin/globulin	0.72	0.05	6.92	0.52	0.02	3.98

**Table 4:** Piroxicam pharmacokinetic parameter values after IV administration as a free drug or as NHs-piroxicam. S = significant statistical differences.

	Piroxicam in Solution	Piroxicam in NHs	Ratio (NHs/Solution)	t-Student	Statistics
**k_el_ (h^−1^)**	0.22 ± 0.03	0.17 ± 0.03	0.78	0.034	S
**V_d_ (l)**	0.44 ± 0.04	0.39 ± 0.03	0.88	0.040	S
**AUC (µg·h/mL)**	3.81 ± 0.76	5.45 ± 0.46	1.43	0.003	S
**Cl (l/h)**	0.10 ± 0.02	0.07 ± 0.01	0.68	0.009	S
**t_1/2_ (h)**	3.21 ± 0.44	4.09 ± 0.59	1.28	0.028	S

## Supplementary Materials

The following are available online at http://www.mdpi.com/1999-4923/10/4/213/s1, Figure S1: FT-IR ATR spectra of HA-Rfv, Figure S2: UV-Vis analysis of HA-Rfv, Figure S3: stability of empty NHs after freeze-drying, Figure S4: stability of empty NHs aqueous suspension, Figure S5: schemes of the syntheses of fluorescent HA and fluorescent NHs, Figure S6: biodistribution of HA and NHs, Figure S7: stability of NHs-piroxicam formulation, Table S1: size and PDI values of NHs-piroxicam formulation.
