# Supplementary material for: Long-Circulating Hyaluronan-Based Nanohydrogels as Carriers of Hydrophobic Drugs
Source: Pharmaceutics. 2018 Nov 3;10(4):213. doi: 10.3390/pharmaceutics10040213 (PMC6320896; doi:10.3390/pharmaceutics10040213)
Supplement: Supplementary file 1 [file pharmaceutics-10-00213-s001.pdf]

Supplementary Materials

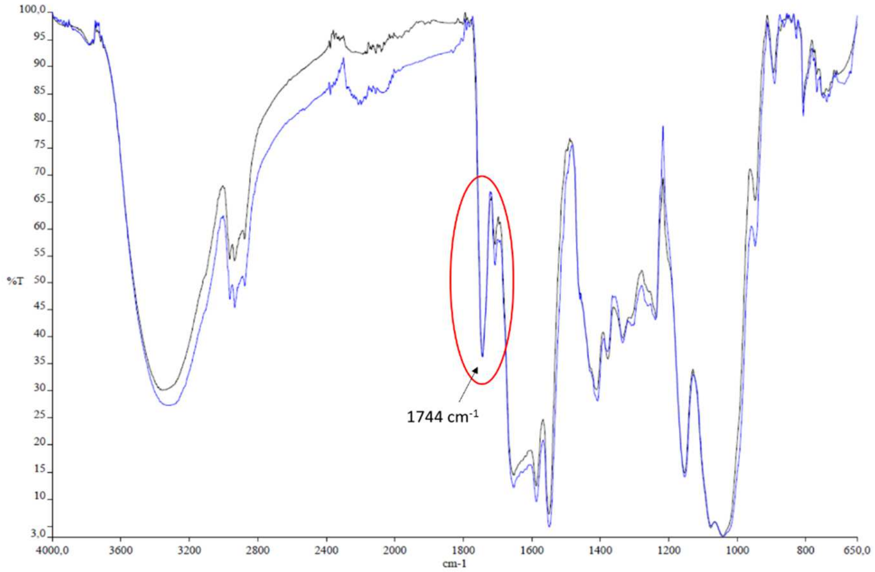

Figure S1: FT-IR ATR spectra of HA-Rfv (black) and HA-Rfv after autoclave treatment (blue). The ester C=O stretching peak at 1744 cm^{-1} is evidenced

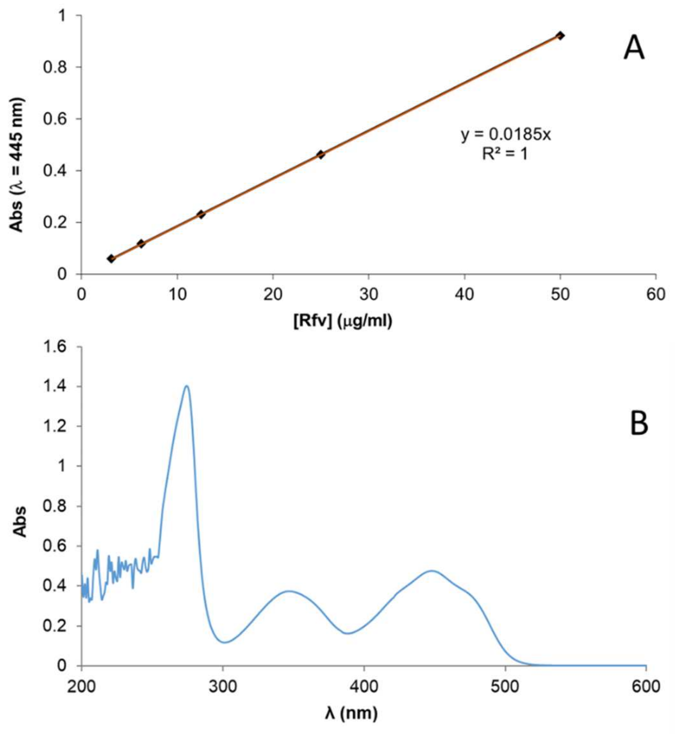

Figure S2: UV-Vis calibration curve of Rfv in DMSO at λ = 445 nm (A); HA-Rfv UV-Vis spectrum at 100 μg/ml in DMSO (B)

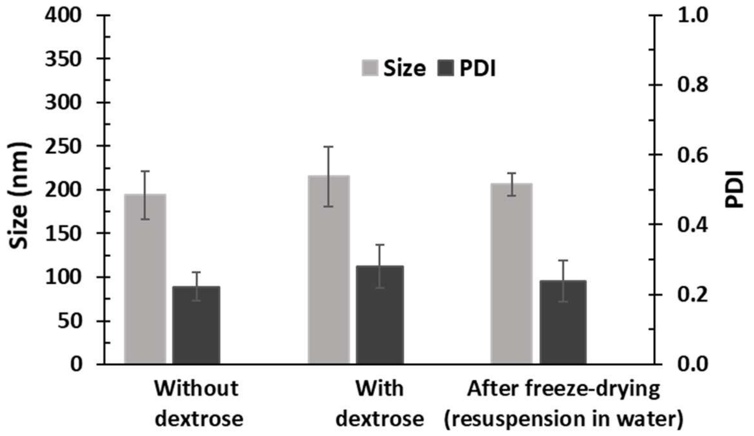

Figure S3: Mean dimensions and PDI of NHs before and after the addition of dextrose (C_f = 1% w/V), and after freeze-drying and re-suspension in water

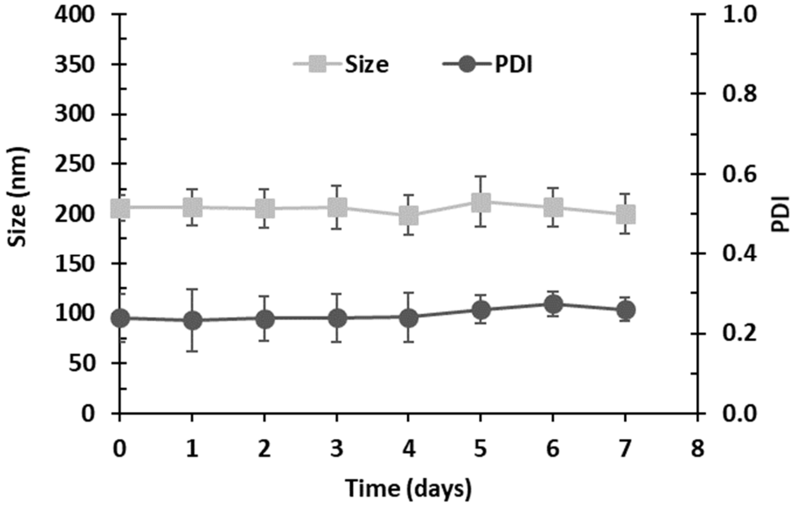

Figure S4: Stability at 4 °C of NHs freeze-dried and re-suspended in water

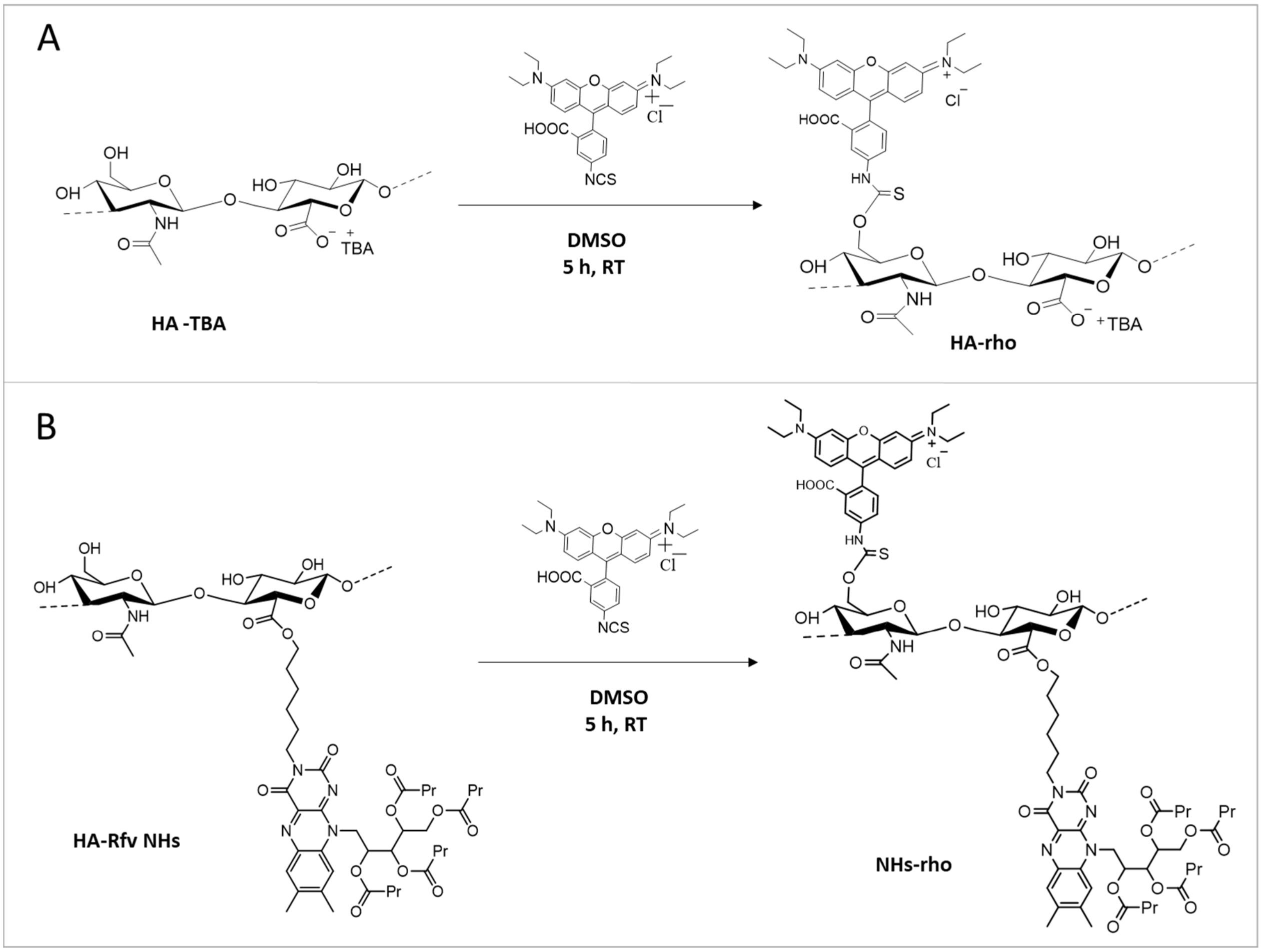

Figure S5: Scheme of reactions of (A) HA-TBA and of (B) HA-Rfv in the form of NHs with Rhodamine B isothiocyanate

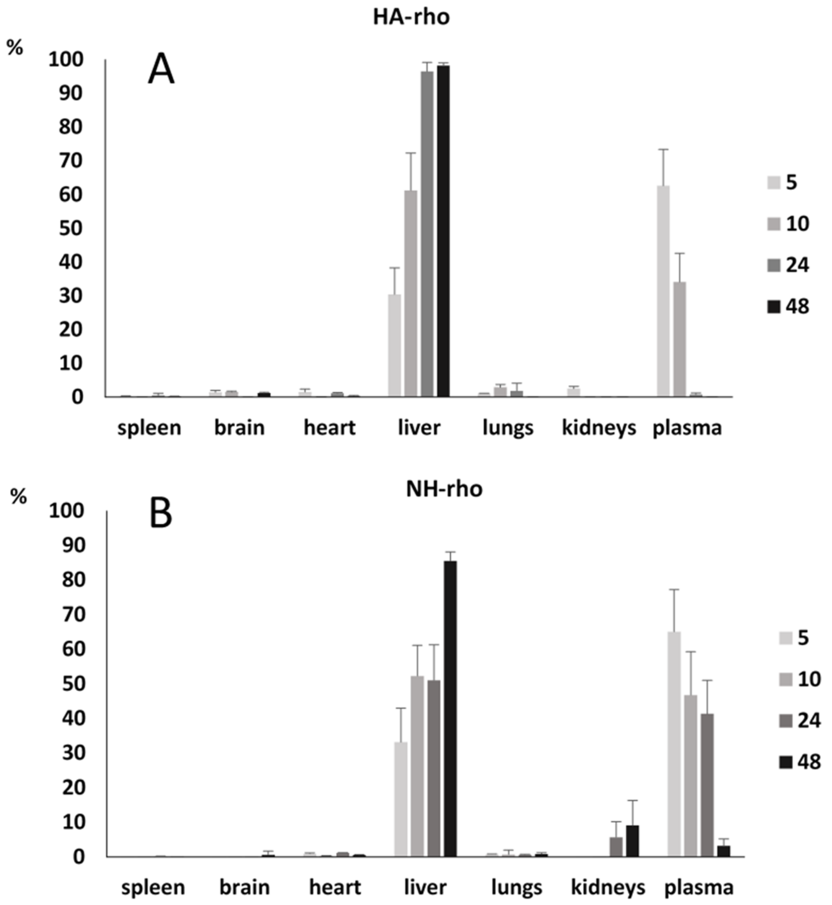

Figure S6: Percentage of accumulation of A) HA and B) NHs at 5, 10, 24 and 48 h of intravenous administration in plasma and analyzed organs

Table S1: Size and PDI of NHs after freeze-drying ad re-suspension in water, and after the addition of glycerol 2.28 % w/V and PBS 0.01 M, pH 7.4

NHs	Size (nm)	PDI
Water (after freeze-drying)	204 ± 20	0.23 ± 0.06
+ Glycerol (2.28 % w/V)	217 ± 20	0.22 ± 0.08
+ PBS 0.01M, pH = 7.4	219 ± 19	0.22 ± 0.04

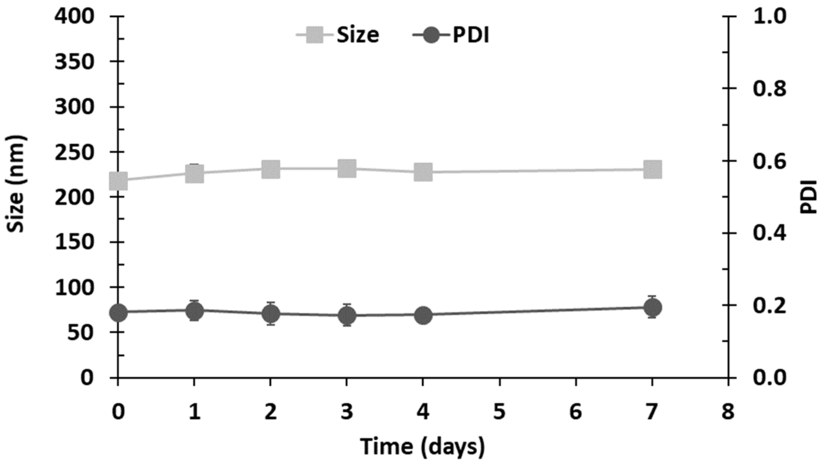

Figure S7: Stability at 4ºC of the piroxicam-loaded NHs formulation in glycerol 2.28% w/V and PBS 0.01 M, pH = 7.4
